# Construction and validation of a prognostic model based on mitochondria-associated endoplasmic reticulum membranes gene signature in LUAD patients

**DOI:** 10.1371/journal.pone.0330722

**Published:** 2025-09-15

**Authors:** Qichen Zhang, Caihong Fu, Shasha Liu, Yue Leng, Ling Duan, Na Wang, Longxia Zhang, Hui Qiao

**Affiliations:** 1 The First School of Clinical Medicine, Lanzhou University, Lanzhou, Gansu, China; 2 Department of Respiratory Oncology, Gansu Provincial Cancer Hospital, Lanzhou, Gansu, China; 3 Department of Pathology, The First Hospital of Lanzhou University, Lanzhou, Gansu, China; 4 Department of Oncology, The First Hospital of Lanzhou University, Lanzhou, Gansu, China; 5 Department of Oncology, The Second Hospital of Lanzhou University, Lanzhou, Gansu, China; 6 Gansu Province Clinical Research Center for Thoracic Tumor, Lanzhou, Gansu, China; The University of Sheffield, UNITED KINGDOM OF GREAT BRITAIN AND NORTHERN IRELAND

## Abstract

**Background:**

Mitochondrial-associated endoplasmic reticulum membranes (MAM) are implicated in various malignancies, but their prognostic value in lung adenocarcinoma (LUAD) remains underexplored.

**Methods:**

The Cancer Genome Atlas (TCGA) and GeneCards databases provided LUAD patient data and MAM-related genes. Differentially expressed genes (DEGs) were identified using the “Limma” package. Enrichment analyses included Gene Ontology (GO), Kyoto Encyclopedia of Genes and Genomes (KEGG), Gene Set Variation Analysis (GSVA), and Gene Set Enrichment Analysis (GSEA). A prognostic risk model based on MAM genes was constructed using univariate, least absolute shrinkage and selection operator (LASSO), and multivariate Cox regression analyses, validated by Receiver Operating Characteristic (ROC) curve analysis. PPI explored intergene relationships, while immune infiltration analysis investigated underlying mechanisms. The Human Protein Atlas (HPA) validated key gene protein expression, and drug sensitivity was analyzed using the Gene Set Cancer Analysis (GSCA) database. Finally, we also performed immunohistochemistry (IHC) staining in the tissue samples.

**Results:**

A prognostic risk model with 3 MAM genes (ERO1A, SHC1, CCT6A) was established from 194 DEGs-MAM. Kaplan-Meier analysis showed significantly longer OS in the low-risk group. Enrichment analyses indicated MAM genes were primarily involved in immune-related pathways. TIMER analysis linked the 3 MAM genes with immune cell infiltration (CD8 + T cells, CD4 + T cells, B cells, macrophages). Expression and prognostic analyses revealed high expression of these mRNAs and proteins in LUAD tissues.

**Conclusions:**

This study constructed a prognostic risk model for LUAD based on 3 MAM genes, revealing a potential link between MAM genes and LUAD, offering new insights into clinical treatment and prognosis.

## 1. Introduction

Lung cancer remains one of the most commonly diagnosed cancers worldwide, with nearly 2.5 million new cases reported in 2022, accounting for approximately 12.4% of all cancer diagnoses. It is also the leading cause of cancer-related mortality, responsible for 1.8 million deaths (18.7%). Lung cancer ranks first in incidence and mortality among men and second among women, with the incidence and mortality rates in men being approximately twice those in women [1]. In China, lung cancer remains the most prevalent and deadliest malignancy, with over 600,000 deaths annually, accounting for 29.71% of all cancer-related deaths. Among non-small cell lung cancer (NSCLC) subtypes, lung adenocarcinoma (LUAD) is the most common globally [2]. Despite significant advancements in the treatment of NSCLC, several antineoplastic modalities are available, including surgery, chemotherapy, radiotherapy, immunotherapy, and targeted therapy. However, due to the numerous adverse effects associated with chemotherapy and radiotherapy, targeted therapy remains one of the most effective treatment approaches [3,4]. Therefore, there is an urgent need for practical prognostic tools to guide personalized treatment strategies for LUAD.

Mitochondrial-associated endoplasmic reticulum membranes (MAM) are regions formed at contact sites between the endoplasmic reticulum (ER) and mitochondria [5]. These contact sites are enriched with enzymes involved in lipid biosynthesis and play an indispensable role in maintaining cellular function and homeostasis [6]. MAM regulate multiple cellular processes, including calcium signaling and dynamics, lipid and lipid intermediate synthesis and transport, autophagy, apoptosis, and energy metabolism. Dysregulation in MAM proteins can disrupt these mechanisms, thereby inhibiting apoptosis and reducing the efficacy of drug therapies [7]. Chemotherapeutic agents that target calcium signaling within MAM have been shown to enhance cancer cells’ responsiveness to therapies that involve Ca^2+^ in their mechanisms [8]. Additionally, MAM has been implicated in the pathogenesis and progression of cardiovascular diseases, endocrine and metabolic disorders, and neurodegenerative diseases, making them significant targets in the treatment of these conditions [9–11]. Notably, numerous studies have linked structural or functional abnormalities in MAM with cancer development and progression. For example, signaling pathways triggered by the displacement of the glycolytic enzyme Hexokinase II (HK2) on MAM have been shown to inhibit tumor cell growth in colorectal and breast cancers [12]. ASK1-mediated MAM stress has been identified as a key mechanism in inducing apoptosis in human ovarian cancer cells [13]. Furthermore, significant ultrastructural abnormalities in MAM, such as changes in density, length, and width between the mitochondrial and ER membranes, have been observed in glioma tissues [14]. Patergnani et al. suggested that intervening in biological processes mediated by certain MAM-related genes might improve the prognosis of patients with malignancies [14].

Although MAM is associated with various malignancies and has been proven to be linked with patient prognosis in several cancers, studies on its role in LUAD remain limited, and the exact mechanisms by which MAM contributes to cancer development are not yet fully understood. Therefore, this study aims to explore the prognostic significance of MAM in LUAD by developing a risk model based on MAM-related genes and to investigate the potential of these genes in guiding clinical treatment strategies for LUAD patients.

## 2. Methods

### 2.1 Acquisition of data

Transcriptomic, clinicopathological, and survival data for LUAD patients were obtained from the TCGA database (https://www.cancer.gov/ccg/research/genome-sequencing/tcga), including 517 tumor samples and 68 normal samples. In addition, the GSE41271 dataset was obtained from the Gene Expression Omnibus (GEO, https://www.ncbi.nlm.nih.gov/geo/) database and was used as an independent external validation cohort. The MAM genes were downloaded from the GeneCards database (https://www.genecards.org/)Immunohistochemistry (IHC) data for LUAD were sourced from the Human Protein Atlas (HPA) database. The flow chart is shown in S1 Fig.

### 2.2 Identification of MAM-Related Differentially Expressed Genes (DEGs)

To identify MAM-related genes, we searched the GeneCards database using the keyword “Mitochondria-associated endoplasmic reticulum membranes.” Differentially expressed genes (DEGs) between LUAD and normal tissues were identified using the “Limma” package [15,16], with a significance threshold of adjust p-value < 0.05 and | log Fold-Change (logFC)| > 1. Visualization of the gene differential expression analysis result was performed using the “pheatmap” and “ggplot2” packages to construct a volcano of the filtered DEGs. Additionally, the online tool Draw Venn Diagrams (https://bioinformatics.psb.ugent.be/webtools/Venn/) was used to obtain differentially expressed MAM-related genes.

### 2.3 Pathway enrichment analysis

Gene ontology (GO) annotation is a widely used method for defining genes and their RNA or protein products, to identify unique biological characteristics from high-throughput transcriptomic or genomic data [17,18]. GO annotations cover 3 aspects of biological content, molecular function (MF), cellular components (CC), and biological process (BP). The Kyoto Encyclopedia of Genes and Genomes (KEGG) is a collection of databases that provides information on genomes, diseases, biological pathways, drugs, and chemical substances [18,19]. We performed GO and KEGG enrichment analyses of MAM-related DEGs using the clusterProfiler, enrichplot, and org.Hs.e.g.,db packages. The “ggplot2” and “GOplot” packages were used for visualization of the results [20–24]. Additionally, we conducted single-gene enrichment analysis using Gene Set Enrichment Analysis (GSEA) software (version 4.2.3), with statistical significance defined by an absolute value of the normalized enrichment score (NES) > 1, p-value < 0.05, and FDR < 0.25. Visualization of the results was performed using the “enrichplot” package [25,26]. We also employed the Gene Set Variation Analysis (GSVA) algorithm from the R package to explore changes in biological processes between two clusters [27].

### 2.4 Construction and validation of the LUAD prognostic model

We utilized the R packages “survival”, “glmnet”, and “rms” to construct and validate the LUAD prognostic model. Initially, univariate Cox regression analysis was employed to identify differentially expressed MAM-related genes associated with overall survival (OS). These genes were further filtered using the least absolute shrinkage and selection operator (LASSO) regression analysis. Finally, multivariate Cox regression analysis was performed to identify prognostically significant MAM-related genes, which were then used to construct the MAM genes prognostic model and calculate risk scores. The specific calculation formula is as follows:


$$\begin{array}{c} \text{risk score}=\sum {\mathrm{\backslash nolimits}}_{i=1}^{n}{exp}_{RNAi}\;*\;{Coef}_{RNAi} \end{array}$$

Based on the median risk score, patients were divided into high-risk and low-risk groups. Kaplan-Meier(K-M) survival analysis was conducted using the “survminer” package to compare survival probabilities and survival times between the high-risk and low-risk groups. To further assess the accuracy of the prognostic model, receiver operating characteristic (ROC) curves were plotted using the “pROC” package [28].

### 2.5 Tumor immune microenvironment analysis

To explore the role of the tumor immune microenvironment in cancer, we utilized the “TIMER” database [29] to evaluate the level of immune cell infiltration and analyze differences in immune cell infiltration between the high-risk and low-risk groups. Additionally, we performed a correlation analysis between MAM-related prognostic genes and key immune checkpoint genes, with the correlation matrix visualized using the “corrplot” package in R.

### 2.6 Protein-protein interaction (PPI) of feature genes and Identification of hub genes

The STRING database (https://string-db.org/) was employed to search for protein interactions of the 3 feature genes, constructing a PPI network and visualizing using Cytoscape software (version 3.8.0). To investigate the potential mechanisms within the PPI network, we selected the top 20 genes with the highest correlation and constructed the regulatory network of these hub genes using Cytoscape software [30]. Additionally, miRNA target gene prediction was performed using the CyTargetLinker plugin, followed by the construction of a miRNA-target gene interaction network [31].

### 2.7 Drug sensitivity analysis

The Gene Set Cancer Analysis (GSCA, http://bioinfo.life.hust.edu.cn/GSCA) platform is an integrated resource for the analysis of genomics, pharmacogenomics, and immunogenomics in cancer. We performed a genomic drug resistance analysis using half-maximal inhibitory concentration (IC50) values from The Cancer Therapeutics Response Portal (CTRP) and Genomics of Drug Sensitivity in Cancer (GDSC) databases [32,33]. The top 30 drugs were visualized using lollipop plots generated with the “ggplot2” package.

### 2.8 Gene expression and prognostic analysis

The protein expression data from the Human Protein Atlas (HPA) database (https://www.proteinatlas.org/) was used to analyze the expression of proteins encoded by differentially regulated genes (DRGs) in lung cancer tissues, validating the protein expression levels of these genes [34]. Additionally, LUAD patients were stratified into high-expression and low-expression groups based on the average expression levels of the 3 genes. K-M survival analysis was then performed to evaluate the differences in OS between these two groups.

### 2.9 Immunohistochemistry analysis of clinical samples

Three pairs of paraffin-embedded tissue block of lung cancer and adjacent normal tissues were obtained from the First Affiliated Hospital of Lanzhou University. Paraffin-embedded tissue sections were deparaffinized using a xylene substitute and rehydrated in a graded ethanol series. Antigen retrieval was performed by heating the sections in EDTA buffer (pH 9.0) at 95°C for 20 minutes. After cooling to room temperature, the sections were washed in phosphate-buffered saline (PBS, pH 7.4) 3 times for 5 minutes each. Endogenous peroxidase activity was quenched by incubating the slides in 3% hydrogen peroxide for 25 minutes in the dark at room temperature. Non-specific binding was blocked by incubating the sections with 3% bovine serum albumin (BSA) for 30 minutes. The slides were then incubated with primary antibodies (dilution 1:200) at 4°C overnight. After washing with PBS, sections were treated with secondary antibodies (1:200) for 50 minutes at room temperature, followed by PBS washes. Detection was performed using 3,3’-diaminobenzidine (DAB) as the chromogen for 30 minutes at room temperature. The reaction was terminated by washing with PBS, and the slides were counterstained with hematoxylin, dehydrated through graded alcohols, cleared in xylene, and mounted. Staining results were visualized and captured using a NIKON ECLIPSE E100 light microscope.

Primary antibodies for CCT6A, ERO1A, and SHC1 were sourced from Proteintech (Wuhan, China), and the secondary antibodies (goat anti-rabbit IgG and goat anti-mouse IgG) were obtained from Servicebio (Wuhan, China).

All lung cancer tissue samples used in this study were obtained with the informed consent of the patients. The study protocol was reviewed and approved by the Ethics Committee of Lanzhou University First Affiliated Hospital, and all procedures were conducted in accordance with the Declaration of Helsinki.

### 2.10 Statistical analysis

Statistical analyses were conducted using R software (version 4.3.2). Comparisons between groups were performed using the Wilcoxon rank-sum test, with results presented as mean ± standard error (SE). The significance of prognostic differences between different sample groups was evaluated using the log-rank test. The relationship between GSVA scores and risk scores, as well as the correlation between gene expression and drug sensitivity, were assessed through Spearman’s correlation analysis. All tests were two-tailed, and a p-value < 0.05 was considered statistically significant.

## 3. Results

### 3.1 Identification and enrichment analysis of DEGs

**Fig 1:**
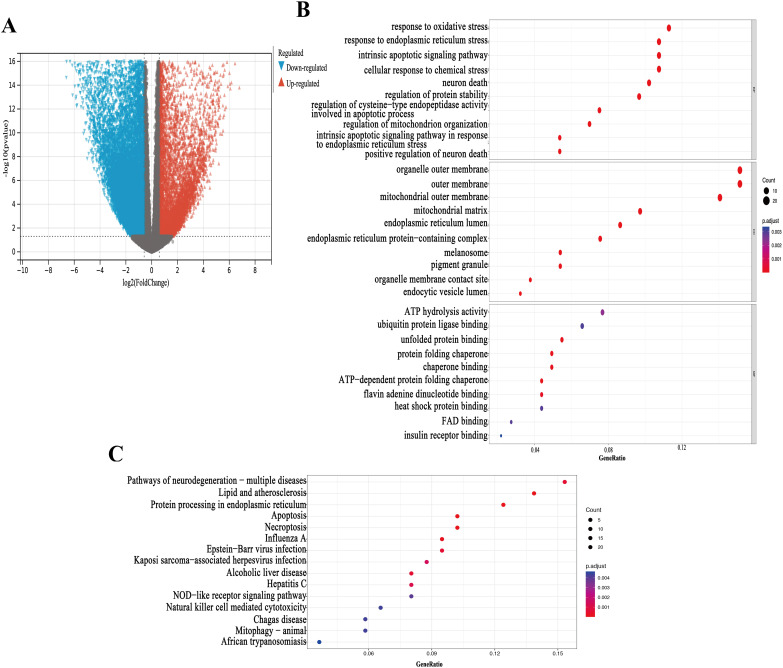
Identification and Enrichment Analysis of DEGs. **A**, Volcano plot showing the differentially expressed genes (DEGs) between LUAD tissues and normal samples in the TCGA dataset; **B**, Gene Ontology (GO) term enrichment analysis of DEGs in LUAD; **C**, Kyoto Encyclopedia of Genes and Genomes (KEGG) pathway enrichment analysis of DEGs in LUAD.

Differential expression analysis identified a total of 22,190 DEGs, with 13,920 genes showing upregulation and 8,270 genes showing downregulation, as illustrated in the volcano plot (Fig 1A). Bubble charts display the top 10 enriched terms for GO-BP, GO-CC, and GO-MF, along with the top 10 enriched KEGG pathways. In the GO enrichment analysis, in terms of BP, the findings were primarily associated with response to oxidative stress, response to endoplasmic reticulum stress, and the intrinsic apoptotic signaling pathway. Regarding CC, the results were mainly linked to functions related to the organelle outer membrane, mitochondrial outer membrane, and mitochondrial matrix. As for MF, significant enrichment was observed in pathways such as ATP hydrolysis activity, ubiquitin protein ligase binding, and unfolded protein binding (Fig 1B). KEGG pathway analysis revealed that the DEGs were mainly enriched in pathways such as neurodegeneration − multiple diseases, Lipid, and atherosclerosis, Protein processing in endoplasmic reticulum, Apoptosis, Necroptosis (Fig 1C).

### 3.2 Identification of MAM genes and screening of prognosis-related genes

**Fig 2:**
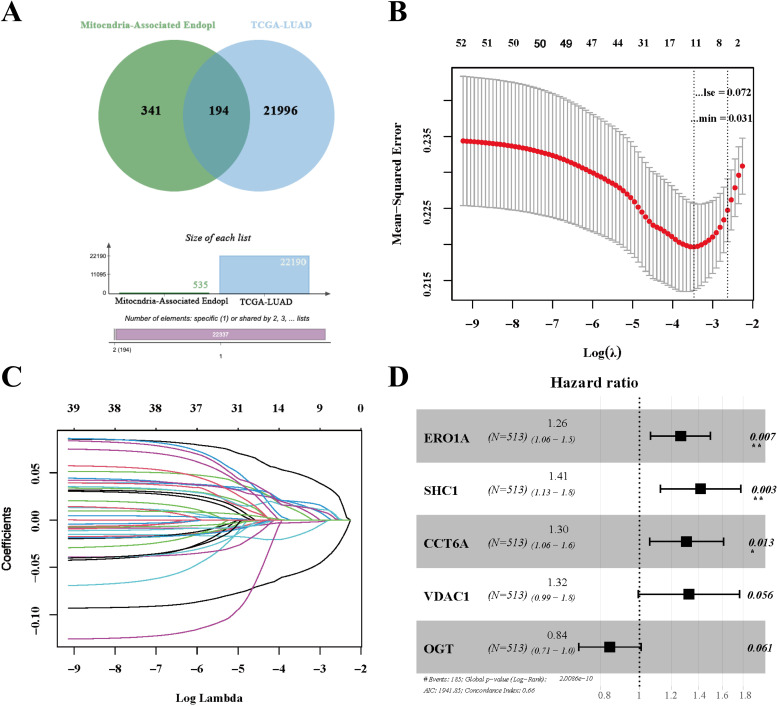
Identification and Prognostic Analysis of MAM-Related Genes in LUAD. **A**, Venn diagram showing the overlap of 194 genes between the 21,996 differentially expressed LUAD genes and 342 MAM genes; **B**, Plot of the log(lambda) sequence generated for the coefficients of MAM genes; **C**, Tenfold cross-validation for variable selection in the LASSO model; **D**, Random-forest plot from multivariable COX regression analysis, the forest plot shows that 3 out of the 5 MAM genes (ERO1A, SHC1, CCT6A) identified through multivariable COX regression are associated with prognosis.

A total of 535 genes associated with the MAM were identified from the GeneCards database. By intersecting the DEGs in LUAD with MAM-related genes, we identified 194 significantly differentially expressed MAM-related genes (Fig 2A). To screen for genes related to patient prognosis from these MAM-related DEGs, univariate Cox regression and batch survival analysis were performed, identifying 39 potential prognostic genes. Subsequently, the LASSO Cox regression analysis, implemented using the “glmnet” package in R, was employed to minimize the risk of overfitting. The optimal λ value, lambda min (0.02595447), was determined, resulting in the identification of 10 prognostic genes (VDAC1, ERO1A, SFTA3, OGT, SHC1, CCT6A, RTN1, GPX8, PAICS, TOMM40) (Fig 2B, C). Finally, multivariate Cox regression analysis further narrowed this list to 3 genes (ERO1A, SHC1, CCT6A), which were used to construct the prognostic risk model (Fig 2D).

### 3.3 Construction and validation of the prognostic risk model

**Fig 3:**
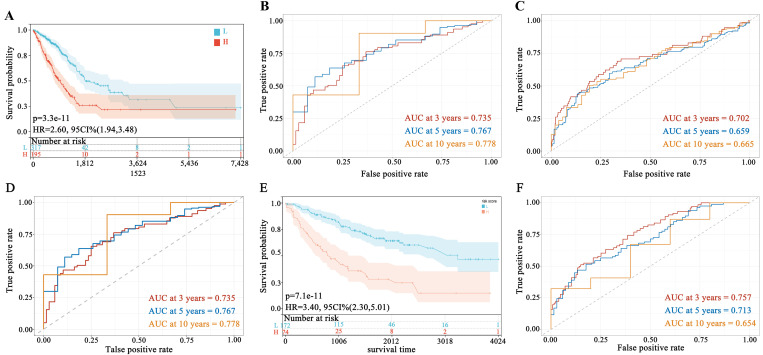
Construction and Validation of the Prognostic Risk Model. **A**, Kaplan-Meier(K-M) survival curve analysis of the MAM-related gene risk score model showing a significant difference in overall survival (OS) between the low-risk and high-risk groups; **B**, ROC analysis validating the model’s performance in predicting the 3-, 5-, and 10-year OS of entire TCGA-LUAD patients; **C**, ROC analysis validating the model’s performance in predicting the 3-, 5-, and 10-year OS of LUAD patients in the testing cohort. **D**, ROC analysis validating the model’s performance in predicting the 3-, 5-, and 10-year OS of LUAD patients in the training cohort. E, K-M survival curve analysis of the MAM-related gene risk score model showing a significant difference in OS between the low-risk and high-risk groups based on GSE41271 dataset. F, ROC analysis validating the model’s performance in predicting the 3-, 5-, and 10-year OS in GSE41271 dataset.

Patients were divided into high-risk and low-risk groups based on the median risk score of 16.7898925 derived from the 3 MAM genes. The log-rank test indicated a significant difference in prognosis between the two groups (p < 0.05). To assess the impact of the high-risk and low-risk scores on prognosis, K-M curves were used to evaluate the prognosis of LUAD patients. The K-M survival curves revealed that patients in the low-risk group had a significantly better OS rate compared to those in the high-risk group (p < 0.05, HR = 2.60, 95% CI [1.94, 3.48]) (Fig 3A). The predictive reliability of the MAM-related prognostic genes was further assessed using ROC curves, revealing that the constructed risk model demonstrated substantial predictive power. In the entire TCGA-LUAD cohort, the AUC values for 3-year, 5-year, and 10-year survival were 0.735, 0.767, and 0.778, respectively (Fig 3B). Additionally, data were split into training and testing cohorts using the “caret” package in R, and the model’s prognostic ability was evaluated through ROC analysis. The AUC values for the testing cohort at 3, 5, and 10 years were 0.702, 0.659, and 0.665, respectively, while those for the training cohort were 0.735, 0.767, and 0.778 (Fig 3C, D). Next, to further evaluate the robustness and generalizability of the prognostic model, we validated its performance using the external independent dataset GSE41271. According to the K-M survival analysis, patients in the high-risk group exhibited significantly worse overall survival compared to those in the low-risk group (p < 0.05, HR = 3.40, 95% CI [2.30 5.01])(Fig 3E). ROC curves assessing the predictive performance of the model in GSE41271. The AUC values at 3, 5, and 10 years were 0.757, 0.713, and 0.654, respectively (Fig 3F). These results demonstrated that the prognostic risk model based on MAM genes exhibited robust predictive performance in LUAD.

### 3.4 GSVA analysis

**Fig 4:**
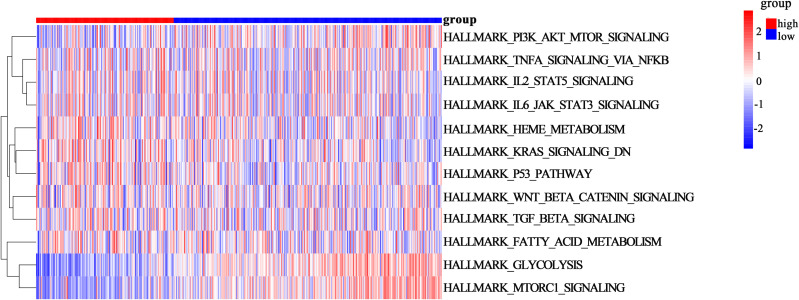
GSVA analysis of high-risk and low-risk groups. Blue represents inhibited pathways, and red represents activated pathways.

To explore the differences in biological processes between the high-risk and low-risk groups, GSVA was conducted on the Hallmark gene sets in LUAD tumors. The results showed that high-risk group pathways significantly enriched in the high-risk group included PI-3K/AKT/mTOR signaling pathway, TNFα signaling via NFkB pathway, IL-2-STAT5 signaling, IL-6/JAK/STAT3 signaling, Heme Metabolism, and TGF-β signaling. In contrast, the low-risk group was mainly enriched in Glycolysis and mTORC1 signaling pathways (Fig 4).

### 3.5 Tumor immune microenvironment analysis

**Fig 5:**
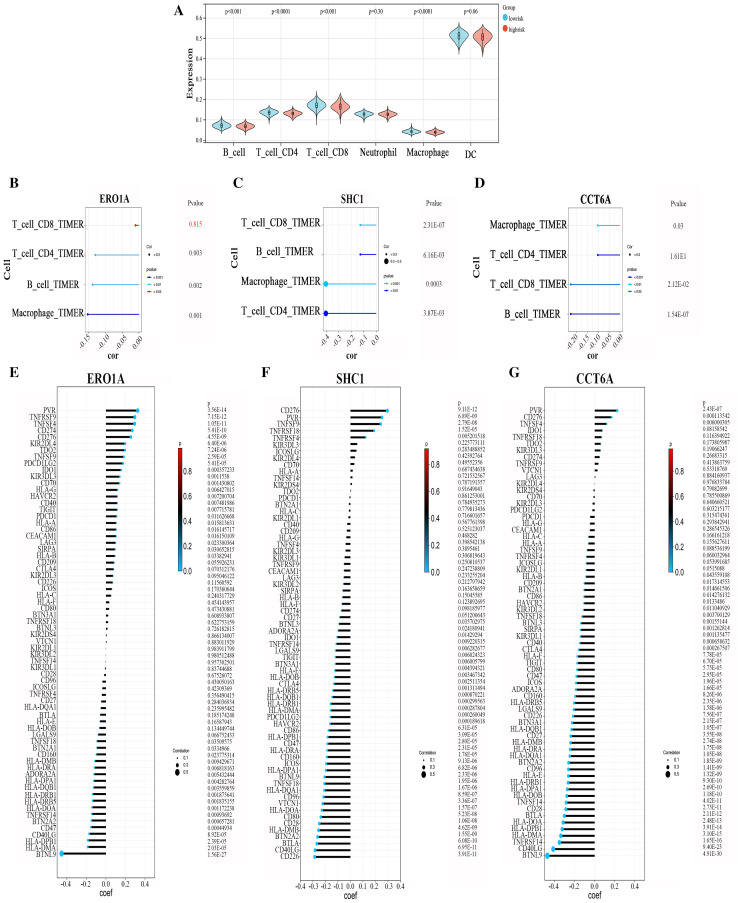
Analysis of the immune microenvironment. **A**, Violin plot showing the differences in the infiltration levels of six immune cell types between high-risk and low-risk groups according to TIMER analysis; **B-D**, Lollipop plots display the correlation between 3 MAM genes and the infiltration levels of 4 immune cell types; **E-G**, Lollipop plots show the correlation between 3 MAM genes and common immune checkpoints.

Given that the GSVA analysis indicated a potential association between LUAD progression and immune-related processes, we investigated the relationship between the risk score and the tumor immune microenvironment. The TIMER algorithm was utilized to assess the infiltration levels of various immune cell subtypes in tumor samples, including B cells, CD4 + T cells, CD8 + T cells, macrophages, dendritic cells, and neutrophils. The TIMER analysis revealed that the infiltration levels of CD8 + T cells, CD4 + T cells, B cells, and macrophages were significantly lower in the high-risk group (p < 0.01) (Fig 5A). Subsequently, we analyzed the correlation between the infiltration levels of these 4 immune cells (CD8 + T cells, CD4 + T cells, B cells, and macrophages) and the 3 MAM genes. The results showed a significant negative correlation for all but the CD8 + T cells with ERO1A expression, which did not show a significant correlation (Fig 5B-D). Additionally, we conducted a correlation analysis between MAM genes and immune checkpoint genes. ERO1A was significantly positively correlated with the expression level of the immune checkpoint gene PVR (p < 0.01) and significantly negatively correlated with BTNL9 expression (r = −0.45, p < 0.01) (Fig 5E). SHC1 showed a significant positive correlation with CD276 (B7-H3) expression (r = 0.30, p < 0.01) and a significant negative correlation with CD226 expression (r = −0.29, p < 0.01) (Fig 5F). Similarly, CCT6A was significantly positively correlated with the expression levels of CD276 (B7-H3) and PVR (p < 0.01) and significantly negatively correlated with the expression levels of immune checkpoints BTNL9 and CD40LG (r = −0.47 and −0.41, p < 0.01,respectively)(Fig 5G).

### 3.6 Expression and prognostic analysis of MAM-related genes

**Fig 6:**
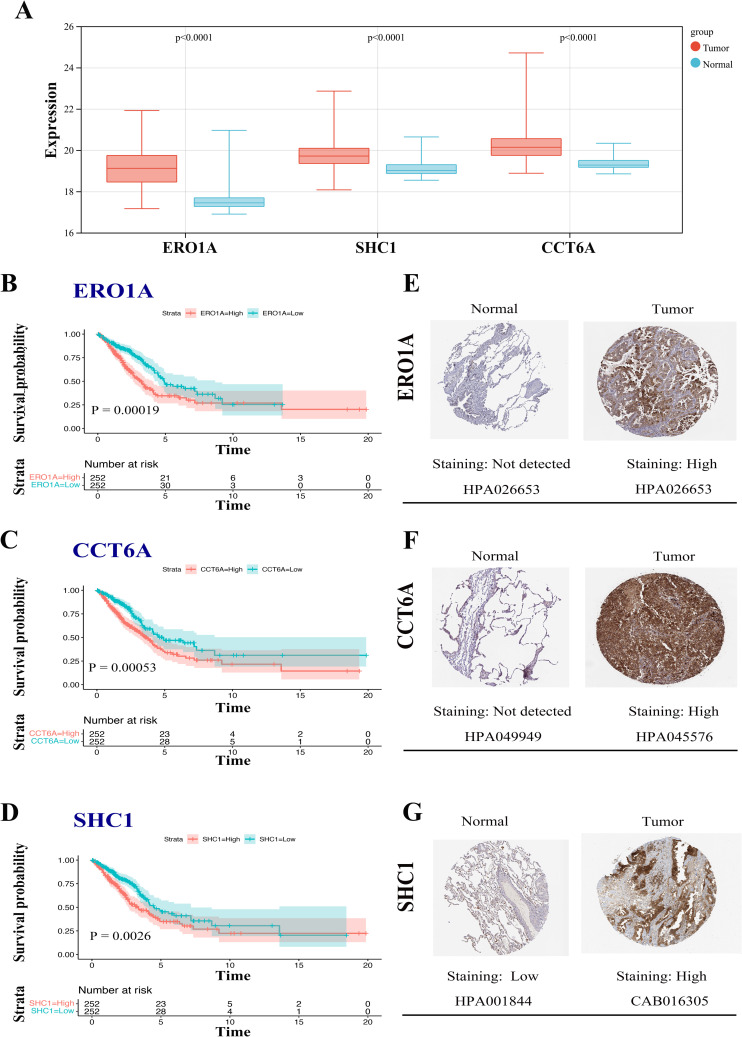
Expression and prognostic value of MAM genes in LUAD patients. **A**, Box plots illustrating the differential expression levels of 3 MAM genes (ERO1A, SHC1, CCT6A) between tumor and normal samples; **B-D**, Kaplan-Meier curves for OS in LUAD patients with high and low expression of ERO1A, CCT6A, and SHC1, respectively; **E-G**, Representative immunohistochemistry images of (E) ERO1A, (F) SHC1, and (G) CCT6A in LUAD and non-cancerous lung tissues from the HPA database.

To validate the prognostic value of the 3 identified MAM genes in LUAD, initially, we examined the expression levels of these risk genes in tumor versus normal tissues. The results indicated that all 3 genes were significantly upregulated in the tumor(P < 0.001) (Fig 6A). Subsequently, the K-M plotter revealed that LUAD patients in the low-expression group for each of the 3 genes had significantly longer OS compared to those in the high-expression group (p < 0.001) (Fig 6B-D). Furthermore, IHC data for these 3 genes were retrieved from the HPA database. The IHC results showed that ERO1A, CCT6A, and SHC1 were significantly overexpressed in tumor tissues compared to normal samples (Fig 6E-G).

**Fig 7:**
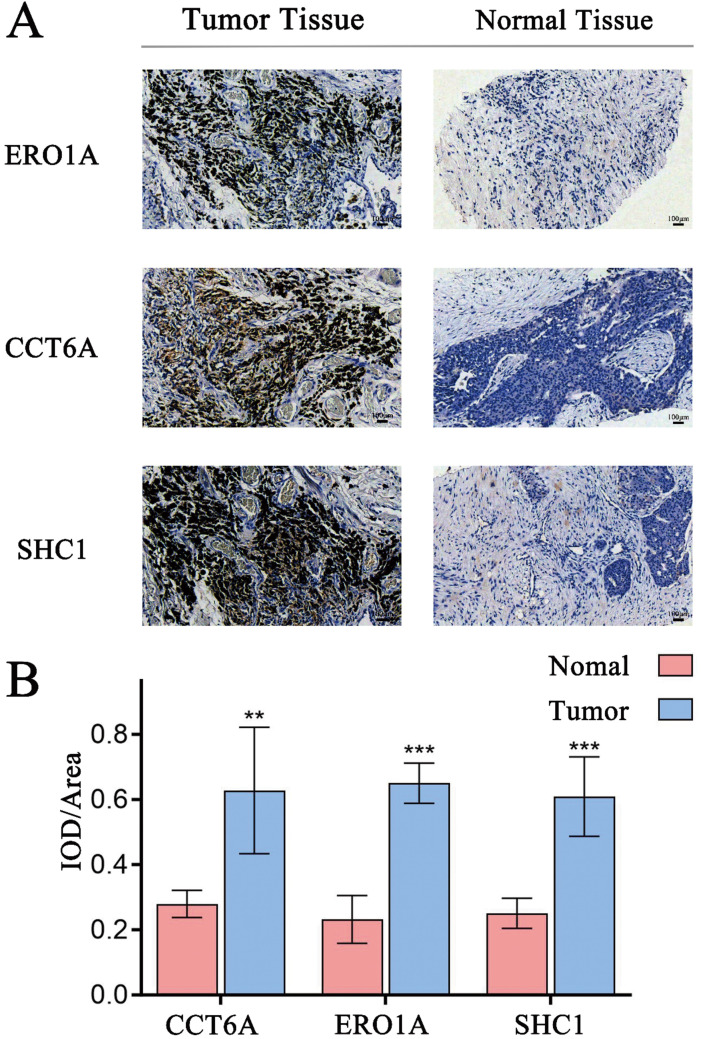
Clinical sample immunohistochemistry results. **A**, The IHC staining results showed that the expression levels of ERO1A, CCT6A, and SHC1 tissues were higher than those in adjacent normal tissues. **B**, Quantitative analysis confirmed the significantly elevated expression of ERO1A, CCT6A, and SHC1 in tumor tissues compared to matched adjacent normal tissues.

To further confirm the relationship between these 3 genes and prognosis, we analyzed 3 pairs of LUAD tissues and corresponding adjacent normal tissues. Immunohistochemical (IHC) staining was performed to compare the expression of the MAM genes (ERO1A, CCT6A, and SHC1) used in the prognostic model. The results demonstrated that all 3 genes were significantly overexpressed in LUAD tissues compared to adjacent normal tissues (Fig 7A-B).

### 3.7 GSEA functional enrichment analysis of the MAM gene prognostic model

**Fig 8:**
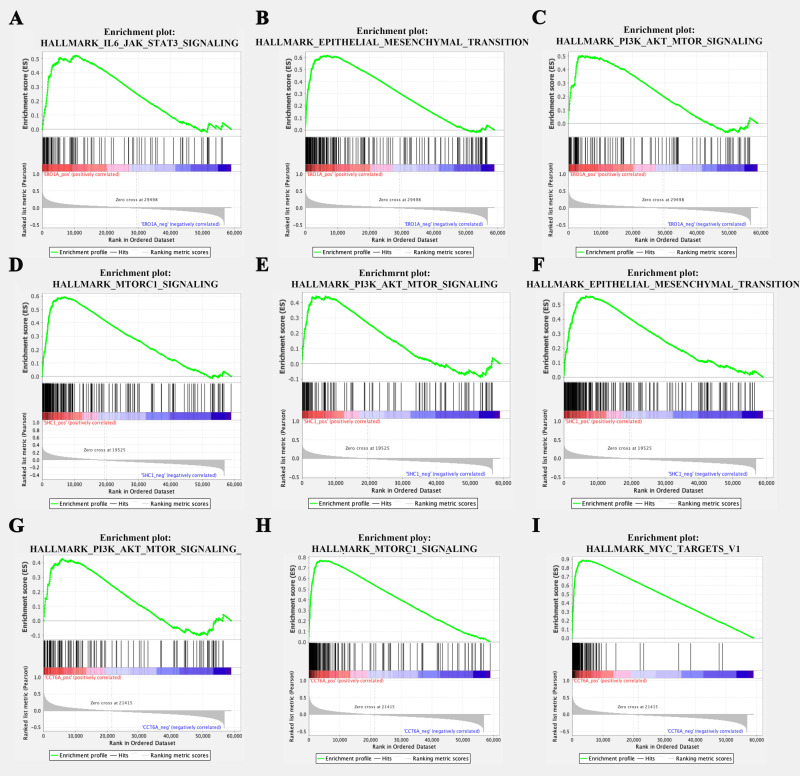
Single-gene GSEA of key genes. **A-C**: GSEA for the ERO1A gene, showing enrichment in (A) IL-6/JAK/STAT3 signaling pathway, (B) Epithelial-mesenchymal transition, and (C) PI-3K/AKT/mTOR signaling pathway; **D-F**: GSEA for the SHC1 gene, showing enrichment in (D) mTORC1 signaling, (E) PI-3K/AKT/mTOR signaling, and (F) Epithelial-mesenchymal transition; **G-I**: GSEA for the CCT6A gene, showing enrichment in (G) PI-3K/AKT/mTOR signaling pathway, (H) mTORC1 signaling, and (I) MYC targets v1.

To explore the potential mechanisms underlying the differences between the high-risk and low-risk groups, GSEA enrichment analysis was performed on the 3 MAM genes (ERO1A, SHC1, and CCT6A). The results showed that the upregulated pathways associated with ERO1A mainly included IL-6/JAK/STAT3 signaling pathway, Epithelial-mesenchymal transition (EMT), and PI-3K/AKT/mTOR signaling pathway (Fig 8A-C). For SHC1, high expression was significantly associated with the upregulation of pathways such as mTORC1 signaling, PI-3K/AKT/mTOR signaling pathway, and EMT (Fig 8D-F). CCT6A high expression was linked to the significant upregulation of pathways including PI-3K/AKT/mTOR signaling pathway, mTORC1 signaling, and MYC targets v1 (Fig 8G-I).

### 3.8 PPI network construction and hub gene identification

**Fig 9:**
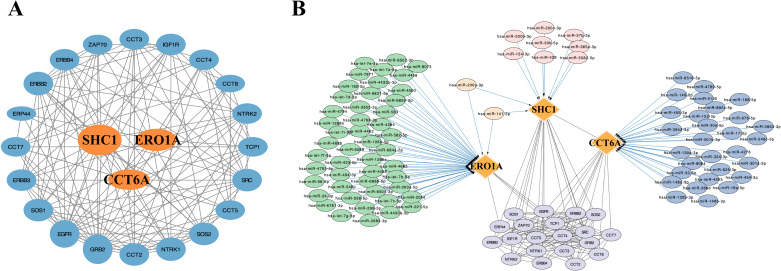
PPI network analysis of hub genes. **A**: Visualization of the PPI network for MAM characteristic genes constructed using Cytoscape and data from the STRING database; **B**: miRNA-target gene interaction network for snRNP characteristic genes, visualized using Cytoscape.

We used the STRING database to identify 20 genes highly correlated with the 3 MAM-related genes. These genes include CCT2, CCT3, CCT4, CCT5, CCT7, CCT8, EGFR, ERBB2, ERBB3, ERBB4, ERP44, GRB2, IGF1R, NTRK1, NTRK2, SOS1, SOS2, SRC, TCP1, and ZAP70, which were constructed a PPI network (Fig 9A). Subsequently, we used the CyTargetLinker plugin in Cytoscape software to predict miRNA target genes and constructed a miRNA-target gene interaction network (Fig 9B). The analysis identified 29 miRNAs related to CCT6A, 50 miRNAs associated with ERO1A, and 10 miRNAs linked to SHC1. Notably, hsa-miR-200a-3p and hsa-miR-141-3p were associated with both ERO1A and SHC1 genes.

### 3.9 Drug sensitivity of LUAD prognosis-related MAM genes

**Fig 10:**
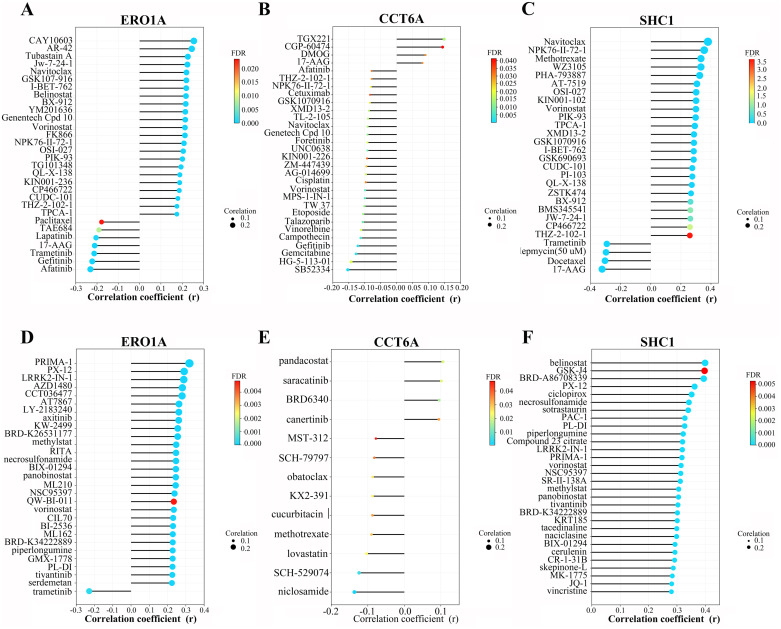
Drug sensitivity analysis of ERO1A, CCT6A, and SHC1 genes based on (A-C) the GDSC database and (D-F) the CTRP database.

Next, we used GSCA to analyze the drug sensitivity of ERO1A, CCT6A, and SHC1 mRNA expression levels in the CTRP and GDSC databases. In the top 30 ranked drugs in the GDSC database (Fig 10A-C), ERO1A expression was positively correlated with the IC50 values of CAY10603, AR-42, Tubastatin A, JW-7-24-1, and others. CCT6A expression positively correlated with the IC50 values of TGX221, CGP-60474, DMOG, and others. SHC1 expression was positively correlated with the IC50 values of Navitoclax, NPK76-II-72–1, Methotrexate, WZ3105, PHA-793887, and others. In the CTRP database (Fig 10D-F), ERO1A expression was negatively correlated with the IC50 value of trametinib but positively correlated with other drugs. Additionally, among the top 30 small molecules in the CTRP database, SHC1 mRNA expression showed a strong negative correlation with IC50 values. For CCT6A mRNA expression, only 13 drugs showed significant correlations, with positive correlations observed for BRD6340, Canertinib, Pandacostat, and Saracatinib, while the other 9 drugs showed negative correlations with IC50 values.

## 4. Discussion

Over the past decade, significant advancements have been made in treating non-small cell lung cancer (NSCLC), including surgery, chemotherapy, radiotherapy, immunotherapy, and targeted therapies. For patients with advanced NSCLC, appropriate targeted therapies have markedly improved survival rates [35].

MAM plays a crucial role in regulating cellular homeostasis and function and they are implicated in various diseases, including neurodegenerative disorders such as Huntington’s disease and Parkinson’s disease, as well as metabolic diseases like non-alcoholic fatty liver disease (NAFLD) and diabetic nephropathy (DN) [36–42]. Numerous studies have demonstrated that MAM was a key regulator in intracellular pathways such as Ca² ⁺ signaling, lipid synthesis, autophagy, and ER stress, contributing significantly to the development and progression of various malignancies [36,43,44]. In this study, we employed bioinformatics approaches to establish a prognostic risk model for LUAD based on 3 MAM genes (ERO1A, CCT6A, and SHC1). Our model demonstrated great predictive performance as evidenced by ROC curve analysis. Furthermore, using data from the HPA database and immunohistochemistry results from our own clinical samples, we found that the protein levels of these genes were significantly reduced in LUAD samples compared to normal tissues. Moreover, this finding was consistent with our transcriptomic analysis results.

ERO1A protein is an endoplasmic reticulum-resident glycoprotein that plays a crucial role in the formation of disulfide bonds [45]. Studies have shown that tumors with overexpressed ERO1A were often found in hypoxic environments, where hypoxic stress can induce immunosuppression by controlling angiogenesis, ultimately leading to resistance to immune checkpoint inhibitor (ICI) therapy [46,47]. In this study, we observed that in LUAD patients within the high-risk group, ERO1A-related pathways, such as the JAK-STAT pathway, were significantly enriched, while immune cell infiltration levels, including CD8 + T cells, CD4 + T cells, and NK cells, were reduced. This finding was consistent with the results reported by Liu et al [48], which demonstrated that ERO1A overexpression is closely associated with the infiltration of immunosuppressive cells, including MDSCs, TAMs, and CAFs. Mechanistically, ERO1A may activate signaling pathways such as JAK-STAT, which suppress T cell proliferation and inhibit cytotoxic T lymphocyte (CTL) responses. It also may prevent the infiltration of CD8 + T cells and NK cells into the tumor microenvironment. Collectively, these effects promote immune tolerance in LUAD patients.

CCT6A, a molecular chaperone protein, plays a critical role in the folding of oncogenic proteins, cellular metabolism, and apoptosis. It has been found that high CCT6A expression promotes the invasion and metastasis of various malignancies, including liver and lung cancers [49–55]. Our functional enrichment analysis revealed that CCT6A overexpression may regulate multiple signaling pathways, including the PI-3K/AKT/mTOR pathway. Previous studies have shown that the PI-3K/AKT/mTOR pathway played a vital role in the development and progression of LUAD by inhibiting apoptosis, promoting cell proliferation, and enhancing angiogenesis [56–58]. Persistent activation of Akt and phosphorylation of mTOR have been observed in more than 50% of NSCLC patient samples and NSCLC cell lines. These alterations were associated with increased invasion and poor prognosis in NSCLC patients [59]. Additionally, it has been reported that inhibiting this pathway using PI-3K inhibitors, such as LY294002, can induce apoptosis in NSCLC cells, enhance CD8 + T cell infiltration, reverse PD-L1-mediated immunosuppression, and increase chemotherapy sensitivity [60,61]. Therefore, we proposed that the CCT6A gene may serve as a key target in LUAD by modulating pathways such as PI-3K/AKT/mTOR, thereby affecting immune cell infiltration in the tumor microenvironment (TME) and playing a critical role in NSCLC progression and drug resistance [62].

SHC1 (SHC-transforming protein 1) is an adaptor protein that plays an essential role in cell signaling, regulation of reactive oxygen species (ROS), stress response, and apoptosis [63–67]. Numerous studies have associated SHC1 with various malignancies, where its overexpression significantly impacts the prognosis of patients with hepatocellular carcinoma (HCC), colorectal cancer, and esophageal squamous cell carcinoma [68–71]. Hudson et al. found that SHC1 promoted metastasis and drug resistance in breast cancer by inducing the EMT process, which was linked to decreased survival rates in breast cancer patients [72]. Notably, high SHC1 expression can also activate the PI-3K/AKT signaling pathway, leading to the suppression of p53 expression and poor prognosis in colorectal cancer patients [73]. Furthermore, our functional enrichment analysis suggested that these key genes were primarily involved in immune-related pathways. It similarly revealed that SHC1 overexpression enriched in several tumor-related signaling pathways, including PI-3K/AKT/mTOR and EMT, and in addition, our results suggested that all 3 genes were involved in immune-associated pathways. A study by James et al. specifically highlighted that SHC activation via the PI-3K pathway was involved in the mitogenic signaling of TCR and B cell antigen receptors and receptor tyrosine kinase families, suggesting that SHC1 may play a significant role in LUAD prognosis by influencing immune cell signaling and promoting cancer cell proliferation [74]. As the internal environment for tumor cell generation and survival, the TME has garnered widespread attention for its impact on tumorigenesis, progression, and metastasis. The TME held significant value in tumor prognosis and evaluating treatment efficacy [75]. In our study, we conducted functional enrichment analysis of the 3 MAM genes. Interestingly, our GSEA analysis identified several TME-related biological processes across all 3 genes, such as IL-6/JAK/STAT3, MYC, and mTORC1 signaling pathways. Given that previous studies have found that modulating MAM may enhance immune cell accessibility to aid cancer treatment [76,77], we focused on the relationship between these 3 genes and immunity.

The TIMER analysis revealed that, compared to the low-risk group, significant differences were primarily observed in B cells, macrophages, CD4 + T cells, and CD8 + T cells. This suggested that immune cell infiltration may play a crucial role in the prognosis of LUAD patients. Furthermore, the correlation analysis between MAM genes and immune checkpoints demonstrated a significant association between these three genes and immune checkpoints.

Both CCT6A and SHC1 genes were found to be significantly positively correlated with the expression levels of B7-H3/CD276. Previous studies have demonstrated that B7-H3/CD276 primarily exerted a co-inhibitory function in tumor immunity and was overexpressed in various malignancies, including NSCLC and breast cancer, where it was closely associated with immune evasion by tumor cells [78–82]. Current research indicated that B7-H3 not only inhibited the proliferation of CD4+ and CD8 + T cells but also suppressed NK cell activity [83,84]. Additionally, Wang et al. discovered that blocking the expression of B7-H3 in human head and neck squamous cell carcinoma (HNSCC) significantly inhibited tumor growth and metastasis [85]. Notably, the expression of B7-H3 is associated with mTORC1 activity, and inhibition of mTORC1 can enhance antitumor immune responses mediated by cytotoxic CD4 + T cells [86]. Our study also found that both CCT6A and SHC1 genes were enriched in the mTORC1 pathway, with a significant reduction in the infiltration levels of CD4+ and CD8 + T cells in the high-risk group. Thus, we hypothesized that CCT6A and SHC1 may influence the expression of B7-H3 through the regulation of the mTORC1 signaling pathway, thereby inducing immune evasion in tumors.

Furthermore, our analysis identified a significant positive correlation between the expression levels of the ERO1A gene and the immune checkpoint molecule CD274. CD274, also known as PD-L1, functions as a ligand for the receptor PD-1, which is primarily expressed on T cells, where it plays a crucial role in suppressing T cell activation [87]. The interaction between PD-1 and PD-L1 is widely recognized as a key mechanism through which tumors evade immune surveillance. Previous studies have established that the expression of CD274/PD-L1 was associated with multiple signaling pathways, including IL-6/JAK/STAT3, PI-3K/AKT/mTOR, JAK/STAT, and WNT, which collectively influenced the tumor immune microenvironment. Consequently, CD274/PD-L1 has emerged as a critical therapeutic target in the immunotherapy of various malignancies [88–93]. For instance, Zhao et al. demonstrated in a study on gastrointestinal stromal tumors (GIST) that PD-1/PD-L1 inhibitors could reduce CD8 + T cell apoptosis by modulating the PI-3K/AKT/mTOR pathway [94]. In our study, TIMER analysis revealed a marked reduction in CD8 + T cell infiltration in high-risk LUAD patients, with ERO1A being predominantly enriched in pathways such as IL-6/JAK/STAT3 and PI-3K/AKT/mTOR. This finding suggested that ERO1A may influence the prognosis of LUAD patients by modulating the immune microenvironment and various signaling pathways, highlighting its potential as a prognostic biomarker for LUAD.

Although this study established a robust MAM-related prognostic model for LUAD and validated its predictive performance using both internal and external datasets, several limitations remain. First, the immunohistochemical validation was conducted using only three pairs of paraffin-embedded LUAD and adjacent normal tissue samples, which may not be sufficient to capture the full heterogeneity of LUAD. Further validation in larger clinical cohorts is warranted. Second, while bioinformatics analyses revealed potential associations between MAM genes and immune-related pathways, no functional experiments were performed to confirm their mechanistic roles in immune modulation or treatment response. Finally, the study relied on cross-sectional transcriptomic data from public databases, limiting the ability to investigate dynamic changes in MAM gene expression and their causal relationships with clinical outcomes or therapeutic efficacy.

In conclusion, this study, from the perspective of MAM, established and validated a prognostic risk model based on the expression of MAM-related genes (ERO1A, SHC1, CCT6A) in LUAD patients, which may provide new clinical insights for improving the treatment and prognosis of LUAD patients. Furthermore, we conducted analyses on immune cell infiltration and immune checkpoint expression levels to determine the potential association between MAM-related prognostic genes and responses to targeted therapy and immunotherapy in LUAD, thereby facilitating personalized patient treatment.

## Supporting information

S1 Fig Flowchart for bioinformatics analysis of TCGA databases. (TIF)
