# Supplementary figures and images for: Construction and validation of a prognostic model based on mitochondria-associated endoplasmic reticulum membranes gene signature in LUAD patients
Source: PLoS One. 2025 Sep 15;20(9):e0330722. doi: 10.1371/journal.pone.0330722 (PMC12435658; doi:10.1371/journal.pone.0330722)

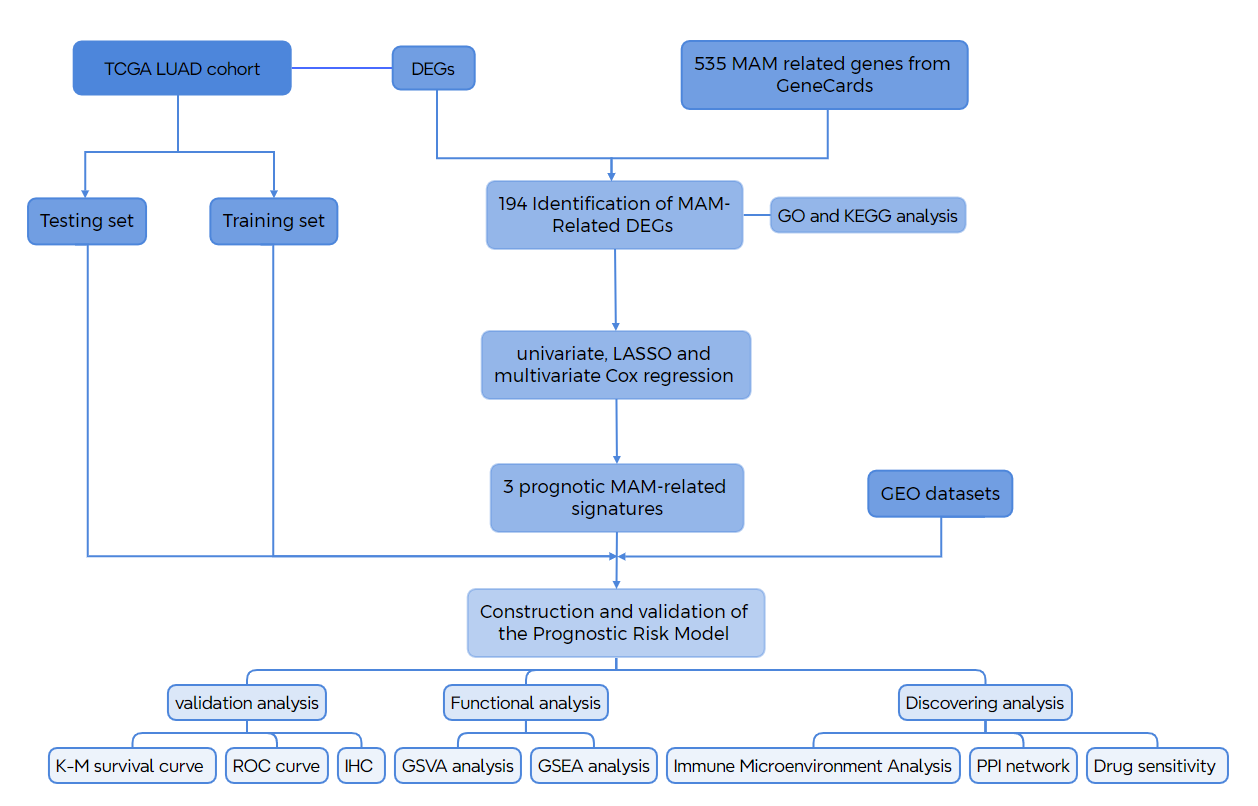

Supplement: S1 Fig — (TIF) [file pone.0330722.s001.tif]
